# Long-Circulating Curcumin-Loaded Liposome Formulations with High Incorporation Efficiency, Stability and Anticancer Activity towards Pancreatic Adenocarcinoma Cell Lines *In Vitro*

**DOI:** 10.1371/journal.pone.0167787

**Published:** 2016-12-09

**Authors:** Mohamed Mahmud, Adriana Piwoni, Nina Filiczak, Martyna Janicka, Jerzy Gubernator

**Affiliations:** Department of Lipids and Liposomes, Faculty of Biotechnology, University of Wroclaw, Wroclaw, Poland; University of South Alabama Mitchell Cancer Institute, UNITED STATES

## Abstract

The incorporation of hydrophobic drugs into liposomes improve their bioavailability and leads to increased stability and anticancer activity, along with decreased drug toxicity. Curcumin (Cur) is a natural polyphenol compound with a potent anticancer activity in pancreatic adenocarcinoma (PA). In the present study, different types of Cur-loaded liposomal formulations were prepared and characterized in terms of size, shape, zeta potential, optimal drug-to-lipid ratio and stability at 4°C, 37°C; and in human plasma in vitro. The best formulation in terms of these parameters was PEGylated, cholesterol-free formulation based upon hydrogenated soya PC (HSPC:DSPE-PEG_2000_:Cur, termed H5), which had a 0.05/10 molar ratio of drug-to-lipid, was found to be stable and had a 96% Cur incorporation efficiency. All Cur-loaded liposomal formulations had potent anticancer activity on the PA cancer cell lines AsPC-1 and BxPC-3, and were less toxic to a normal cell line (NHDF). Furthermore, apoptosis-induction induced by Cur in PA cells was associated with morphological changes including cell shrinkage, cytoplasmic blebbing, irregularity in shape and the externalization of cell membrane phosphatidylserine, which was preceded by an increase in intracellular reactive oxygen species (ROS) generation and caspase 3/7 activation. Because the liposomal formulations tested here, especially the H5 variant which exhibited slow release of the Cur in the human plasma test, the formulation may be stable enough to facilitate the accumulation of pharmacologically active amounts of Cur in target cancer tissue by EPR. Therefore, our formulations could serve as a promising therapeutic approach for pancreatic cancer and other cancers.

## Introduction

Pancreatic adenocarcinoma (PA) is one of the five most common causes of cancer-related mortality worldwide, with 1- and 5-year survival rates of 25% and less than 5%, respectively [1–2]. The poor survival rates of PA patients has been substantially unchanged over the past 30 years, despite advances in molecular biology, pathological classification, as well as clinical therapies including surgical resection, radiotherapy and chemotherapy [2–3]. Gemcitabine has become a standard chemotherapy for patients with locally advanced and metastatic pancreatic cancer since 1997. However, the level of clinical benefit response of gemcitabine is meager (less than 6 months) [3–4]. The main reason for the ineffective treatment of PA is the presence of highly fibrotic stromal components, including abundant collagen and hyaluronan, which are not found in most other solid tumors [5–6]. Other reasons are connected with a lack of presentation of cancer-specific symptoms, resulting in the inability to diagnose at an early stage (the disease is usually only manifest at the metastatic stage, where it has already spread to other organs) and resistance to the treatment [7]. Consequently, an enormous amount of research needs to be done in order to improve the survival rate, whether by increasing the efficacy of existing drugs in combination with other cytotoxics, or finding a suitable drug-carrier that shows a significantly improved pharmacological effect.

Preclinical studies using bioactive compounds such as curcumin, vitamin E, D, gingerol, crocetin and triterpenoids, in combination with standard chemotherapy are currently in progress, with the aim of improving existing treatments and to discover more effective ways to treat PA [8,9]. Curcumin (Cur, diferuloylmethane) is a yellow phytochemical substance that is derived from the dried rhizome of the East Indian plant, *Curcuma longa* (turmeric), popularly known as ‘‘curry powder”. Numerous preclinical studies over the last two decades have demonstrated that Cur possesses potent anti-inflammatory, anti-oxidant, anticancer properties with low cytotoxicity. It has been used as a chemopreventative agent in a wide variety of different cancers, including leukemia and lymphoma, gastrointestinal cancers (colon, gastric, hepatic, pancreatic), genitourinary cancers (prostate), breast cancer, ovarian cancer, head and neck squamous cell carcinoma, lung cancer, melanoma, neurological cancers and sarcoma [4,10].

Cur has a variety of effects on several signaling pathways which play an important role in antitumor activity. It suppresses nuclear transcription factor (NF-κB) activation, cell-cycle regulators (cyclin D1), cytokine mediators (IL-1 and IL-8) and enzymes (COX2); and induces cytochrome c release, as well as the caspase activation pathway (caspase-8, 3/7 and 9) and the tumor suppressor pathway (p53), which lead to PA cell death [4,9]. Out of all these molecules, NF-κB, which has antiapoptotic properties in PA, has been considered to be the main target of Cur in PA and other cancer cells [10].

Apart from its great medicinal benefits, Cur has been approved as a safe compound by the World Health Organization and the US Food and Drug Administration (FDA). However, the extremely poor aqueous-solubility and poor bioavailability of Cur have hampered its clinical uses for cancer treatment [11]. Therefore, nanoscale drug delivery systems including, liposomes [12] liquid crystal [13] solid lipid nanoparticles [14] nanoemulsion [15], and phospholipids complex [16], have been used as means to overcome these disadvantages. In particular, liposomes have been considered as a promising drug nanocarrier vehicle for Cur and many other chemotherapeutic drugs due to the potential to improve the bioavailability, pharmacokinetic and pharmacodynamics of these drugs. In order to treat PA, special approaches must be taken into consideration. First of all, the PA cancer mass is very heterogeneous, with the highest amount of extracellular matrix of almost any tumor, which markedly impairs drug accumulation [3]. For PEGylated particles in the size of about 100 nm, extravasation is observed in inflamed tissues due to the leaky vasculature, especially in the case of fast-growing tumors. This so-called EPR effect (enhanced permeability and retention) enables better accumulation of the drugs in many tumors [17–21]. However, in the case of PA, the liposomal drug accumulating in cancer tissue via the EPR effect can be lower due to the presence of the extracellular matrix. Therefore, repeated doses of liposomal Cur should be taken in consideration in order to overcome this limitation. Multiple dosing influences increased drug accumulation within tumors, leading to tumor saturation to a greater extent than in the case of a simple injection. The increased activity of anticancer agents after multiple dosing of bleomycin has been observed by others in the 4T1 murine breast cancer model, where only repeated injection of the drug-loaded liposomes containing bleomycin resulted in tumor. Therefore, we decided to prepare nanometer-scale drug carriers that could be used in metronomic therapy, with the objective of achieving increased levels of curcumin inside the tumor tissue via the EPR. This approach can be made for such formulated curcumin or in combination with the liposomal epirubicin which was previously prepared in our laboratory [22]. The combination experiments are ongoing in our laboratory giving an encouraging results. In order to achieve this goal at least two factors must be fulfilled: the nanocarrier must be long-circulating, to accumulate in a reasonable amount within the cancer tissue and, secondly, the nanocarrier should retain Cur for an extended period in the circulation, without any appreciable loss from within the liposomes.

In the present study, liposomes were used as a drug delivery system in order to improve the stability, bioavailability and anticancer activity of lipophilic Cur. So far, a number of liposomal formulations of Cur have been reported by others groups. Most of them were based on the classical negatively-charged, non-long circulating liposomes composed from the lipids being in a liquid-crystalline state at 37°C (DMPC, DOPC) [12,23,24]. This approach facilitates Cur encapsulation and increases drug bioavailability but does not allow the benefit from an extended circulation time and, therefore, limits the accumulation of such liposomes in the cancer tissue via the EPR effect. Additionally, charged liposomes are cleared much faster by the mononuclear phagocyte system (MPS) compared to their neutral counterparts [25–27]. Since PEGylated liposomes composed from phospholipids having saturated fatty-acids (such as HSPC) exhibit a long circulation-time and can efficiently deliver their payload to the cancer tissue via EPR effect [28,29], this type of liposomal formulation was selected as a liposome base for use in our studies. Several Cur liposomal compositions were characterized based on a number of biophysical parameters, including the size, shape, zeta potential, optimal drug-to-lipid ratio, incorporation efficiency and in vitro stability and cytotoxicity of the selected formulations. Finally, ROS generation, caspase 3/7 activation and morphological changes on PA cell lines were assessed when exposed to the best liposomal formulation in term of above parameters, namely the H5 formulation. To the best of our knowledge, we are the first to report that lower drug-to-lipid ratio of lipophilic Cur to liposomes increased its stability in plasma and bioavailability in a PA cell model potentiating Cur activity. After performing experiments in 50% human plasma and in vitro studies, we hypothesize that Cur molecules in liposomes with higher drug-to-lipid ratios form clusters enabling drug leakage in circulation and decrease Cur bioavailability after liposomes internalization.

## Materials and Methods

### Materials

Hydrogenated soya phosphatidylcholine (Phospholipon^®^ 90H, HSPC), soya phosphatidylcholine or (Phospholipon^®^ 90G, SPC), 1,2-distearol-sn-glycero-phosphoethanolamin-N-(poly[ethylene glycol]2000) (DSPE-PEG_2000_) were purchased from Lipoid GmbH (Ludwigshafen, Germany). Cholesterol (Chol) was purchased from Northern Lipids (Vancouver, BC, Canada). Curcumin 95% was purchased from Apollo Scientific Ltd (Stockport, UK). Sodium Chloride was purchased from Avantor Performance Materials (Gliwice, Poland).

### Preparation of curcumin-loaded liposomes

Cur-loaded liposomes were formulated using the extrusion technique. In brief, lipids and Cur were dissolved in chloroform to obtain stock solutions at 10 and 5 mg/mL, respectively. Cur was mixed together with 40 mg of lipid in borosilicate glass tubes at a range of molar ratios (see Table 1). Chloroform was removed from the samples via evaporation under a stream of nitrogen-gas and the resultant lipid film was dissolved in a mixture of cyclohexane and methanol (99:1, v/v). The samples were frozen in liquid nitrogen and freeze-dried for 8 hours at low pressure using a Savant Modulyo apparatus (Thermo Fisher Scientific, Waltham, MA, USA). The lipid films were hydrated by addition of 1.5 ml of 150 mM NaCl at 64°C, in a water bath, with gentle mixing. The liposomal suspensions were finally sonicated in a water bath sonicator for 8 minutes at 64°C. The newly-formed multilamellar vesicles (MLVs) were extruded 10 times through Nucleopore plycarbonate filters (Whatman, Maidstone, UK) with pore sizes of 400 and 100 nm, respectively, using a Thermobarrel Extruder (10 ml Lipex extruder, Northern Lipids, Canada) to obtain large unilamellar vesicles (LUVs). The extruder was maintained at 64°C throughout the liposome extrusion procedure.

**Table 1:** Liposome compositions used in the study.

Formulations [Molar-Ratios]		HSPC	SPC	Chol	DSPE-PEG_2000_	Cur
Liposome type	Code					
C-HSPC-L						
	H5	9.0	-	-	0.5	0.5
	H10	8.5	-	-	0.5	1.0
	H12	8.3	-	-	0.5	1.2
	H15	8.0	-	-	0.5	1.5
C-HSPC-Cho-L						
	HCh10	5.5	-	3.0	0.5	1.0
C-SPC-Cho-L						
	SCh5	-	6.0	3.0	0.5	0.5
	SCh10	-	5.5	3.0	0.5	1.0
	SCh12	-	5.3	3.0	0.5	1.2

### Characterization of curcumin-loaded liposomes

#### Determination of the mean diameter size, polydispersity and zeta potential of curcumin loaded liposomes

Cur-loaded liposomes were diluted to a total lipid concentration (10 μM in 150 mM NaCl) before measuring the mean diameter size, polydispersity (PDI) and zeta potential. All determinations were recorded at room temperature (25°C) using a Zetasizer Nano ZS (Malvern Instruments, Malvern, UK).

#### Morphology of curcumin-loaded liposomes

Transmission electron microscopy (TEM) (Tesla BS 540 JEOL 100) was used to observe the microstructure and morphology of Cur-loaded liposomes using a negative-staining method. A diluted drop of Cur-loaded liposomes was placed on a copper grid and dried at room temperature. This was followed by staining with 2% of ammonium molybdate and dried at room temperature as well. The sample was subsequently observed via TEM.

### Determination of incorporation efficiency of curcumin-loaded liposomes

Non-incorporated drug-crystals were separated from the Cur-loaded liposomes during the liposome extrusion procedure (only Cur-loaded liposomes can pass through Nucleopore polycarbonate filters). Additionally, the samples were centrifuged and then collected to ensure the absence of any free Cur. Liposome samples (50 μL) were taken before extrusion (initial) and after centrifugation. The lipid concentration was determined by the ammonium ferrothicyanate assay [30] on a Varian Cary^®^ 50 UV-Vis Spectrophotometer (Varian, Ltd., Victoria, Australia). The concentration of Cur in the liposomes was determined photometrically at λ = 425 nm on the same spectrophotometer after the Cur-loaded liposomes were dissolved in methanol. The incorporation efficiency (IE %) was calculated according to the following equation:

$$\text{IE}(\%)=\frac{\text{Cae}(\text{mg}/\text{mL})/\text{Lae}(\text{mg}/\text{mL})}{\text{Cbe}(\text{mg}/\text{mL})/\text{Lbe}(\text{mg}/\text{mL})}\times 100$$

where Cae and Lea represent the concentration of Cur and lipids after extrusion, ae, and Cbe and Lbe represent the initial concentration of Cur and lipids before extrusion, be.

The amount of Cur was calculated from the appropriate calibration curve, and was in the range of 1–6 μg/mL, R^2^ = 0.999.

### Stability of curcumin-loaded liposomes

The stability studies of the selected Cur-loaded liposome formulations were performed at 4°C and 37°C, with storage times of 45 days and 48 hours, respectively. After the respective storage periods, the samples were centrifuged at 11,000 rpm for 5 min in order to observe the appearance of non-incorporated Cur (crystals) by visual inspection. Also, liposome samples (50 μL) from the supernatants were collected at 4°C at different intervals (day 15, day 30 and day 45) and at 37°C at different intervals (0, 0.5, 2, 4, 6, 12, 24 and 48 hours). The concentrations of Cur and the lipid were measured in the collected liposomal samples fraction and compared with the initial concentration of Cur and lipids, as described above for the determination of IE (%).

### In vitro release of curcumin from liposomes in the presence of human plasma

The selected Cur-loaded liposome formulations were diluted to obtain a total lipid concentration of 4 mM and then mixed with fresh human plasma (1:1, v/v). The liposomes were incubated in an incubator (Binder, Tuttlingen, Germany) at 37°C for 48 hours. Samples (200 μL) at 0, 0.5, 2, 4, 6, 12, 24 and 48 hours were placed on Sepharose CL 4B minicolumns (5.5 × 70 mm) and subsequently protein-bound Cur was separated from Cur-loaded liposomes. The Cur and the lipid concentrations were measured in the collected liposomal sample fraction and compared with the initial concentration of Cur and lipids, as described above for the determination of IE (%).

### Pancreatic cancer and normal cell culture

Human pancreatic cell lines AsPC-1 (provided by the Institute of Immunology and Experimental Therapy, Wroclaw, Poland) and BxPC-3 (purchased from ATCC) were cultured in RPMI 1640 medium (Lonza, Warsaw, Poland) supplemented with 10% fetal bovine serum, 2 mM glutamine (Life Technology, Warsaw, Poland), 100 U/mL penicillin, 100 μg/mL streptomycin, and 25 μg/mL amphotericin B (Polfa Tarchomin, Warsaw, Poland). The normal human dermal fibroblast cell line (NHDF, Lonza) was cultured in Minimum Essential Medium Eagle (EMEM) Alpha Modifications medium (Lonza) supplemented with 10% fetal bovine serum and 2mM glutamine, 100 U/mL penicillin, 100 μg/mL streptomycin, and 25 μg/mL amphotericin B. The cells were cultured in a 37°C incubator in a humidified atmosphere containing 5% CO_2_.

### Determination of in vitro cytotoxicity

#### MTT assay

Cytotoxicity was determined using the MTT (3-[4,5-dimethylthiazol-2-yl]-2,5-diphenyltetrazolium bromide) assay. In brief, AsPC-1, BxPC-3 and NHDF cells were seeded in triplicate into 96-well culture plates at 10^4^ cells/well and incubated at 37°C in a humidified atmosphere and 5% CO_2_ overnight. Cells, in triplicate wells, were treated in a dose concentration-dependent manner with free Cur, free liposome (lipo) and different Cur-loaded liposome formulations including (H5, H10, SCh5 and SCh10), and incubated for 72 hours. Subsequently, the medium was carefully removed from the wells, followed by addition of 50 μL of dye solution (0.5 mg/mL of MTT salt in culture medium) (Sigma-Aldrich, Poznan, Poland) and incubated at 37°C for 4 hours. The absorbance of the samples was calorimetrically measured at 550 nm with a reference wavelength of 630 nm on a microplate reader UVM 340 (Biogenet, Poland). The untreated control was normalized to 100% for each assay, and treatments were expressed as the percentage of control.

#### ATP assay

Cell cytotoxicity was determined based on the level of intracellular adenosine 5′-triphosphate (ATP). The assay was performed according to the manufacturer’s instructions (Kit CellTiter-Glo^®^ luminescent cell viability assay, Promega, Warsaw, Poland; Cat. no. G7571). In brief, AsPC-1 and BxPC-3 cells were seeded and treated as previously described, except that they were seeded into white opaque 96-well culture plates (Scholagene, Krakow, Poland) in this instance. After 72 hours, the medium was carefully removed from the wells, followed by addition of 100 μL of medium. The cells were then lysed with 100 μL of lysis buffer and the plate shaken for 2 minutes on an orbital shaker in order to induce cell lysis. The plate was incubated for 10 min at ambient temperature in the dark. The luminescence was measured for 1 second per well using a Microlumat LB 96 P microplate luminometer (EG&G Berthold, Wildbad, Germany). The relative light units (RLUs) were normalized against control (100% untreated cell), and treatments were expressed as the percentage of control.

### Determination of intracellular reactive-oxygen species levels

Intracellular reactive oxygen species (ROS) generation was monitored by using the 2',7'-dichlorodihydrofluorescein diacetate (H_2_DCFDA) assay. In brief, AsPC-1 and BxPC-3 cells were seeded as previously described, but into black 96-well culture plates (Scholagene). Cells were treated in a time-dependent manner with Cur in a selected formulation (H5) and free Cur, both at a concentration equivalent to the IC50 of Cur; and incubated for the selected time intervals of 1, 3, 6 and 24 hours at 37°C. The medium was then removed from the wells, followed by washing with Dulbecco’s Phosphate Buffered Saline with D-Glucose and Sodium Pyruvate (DPBS, Lonza). 100 μL of H_2_DCFDA solution (10 μM in DPBS) (Life Technology, Warsaw, Poland) was added and the plate incubated for 30 minutes at 37°C. The H_2_DCFDA was removed and 200 μL of DPBS was added to each well. The fluorescent intensity was recorded by excitation at 498 nm and emission at 525 nm on a Varian Cary Eclipse Fluorescence Spectrophotometer (Varian, Ltd., Victoria, Australia). ROS level was expressed as the percentage of control (100%).

### Determination of enzymatic caspase 3/7 activity and cell morphology

Caspase 3/7 activity was monitored in a real-time manner using an Incucyte^™^ Live-Cell Imaging System (Essen BioScience, Hertfordshire, United Kingdom). The assay was performed according to the manufacturer’s instructions (CellPlayerTM 96-well kinetic Caspase 3/7 reagent kits). In brief, AsPC-1 and BxPC-3 cells were seeded as previously described, in triplicate, into 96-well culture plates and treated as described for the ROS assay. Subsequently, cells were stained with DEVD-Nuc- View 488 caspase-3 substrate at a final concentration of 5 μM. Real-time green-fluorescence images were taken at two-hour intervals. Phase-contrast images were taken alongside the green fluorescence images to determine cell morphology. Data analysis in both instances was conducted using the IncuCyte ZOOM 2016A software.

### Detection of apoptosis using annexin V and propidium iodide staining

Apoptotic and necrotic cells were distinguished on the basis of Annexin V-fluorescein isothiocyanate and propidium iodide (Annexin V-FITC/PI) double-staining. The staining was performed according to the manufacturer’s instructions (BD Pharmingen^™^, Warsaw, Poland). In brief, AsPC-1 and BxPC-3 cells were seeded into 24-well culture plates (Scholagene) at 5 × 10^4^ cells per well and incubated at 37°C in a humidified atmosphere of 5% CO_2_ overnight. The cells were treated as previously described in the ROS assay. 30 min prior of examination, 52 μL of 10% H_2_O_2_ was added as a positive control of apoptosis. The medium was carefully removed from the wells, followed by washing twice with PBS. 200 μL of 1X Annexin Binding Buffer was added to each well. Subsequently, cells were stained with 2, 2 and 1 μL of Annexin V-FITC, PI (50 μg/ml in PBS) and 10X Hoechst 33342 (in Annexin Binding Buffer), respectively. The plate was incubated for 10 min at ambient temperature in the dark. The cells were monitored under a fluorescence microscope Nikon Eclipsce 90i (Nikon, Tokio, Japan) using a 40x PlanApo lens.

### Statistical analysis

The data are expressed as the means ± standard error. Statistical analyses were done using GraphPad Prism (software version 7.0. VA). Differences between untreated (Control) vs. treated were determined by the unpaired student *t*-test. A *p* value of < 0.05 (*) or < 0.001 (**) was considered as statistically significant. The 50% inhibitory concentrations (IC50 values) were also calculated using GraphPad Prism.

## Results

### Liposome characterization

**Fig 1:**
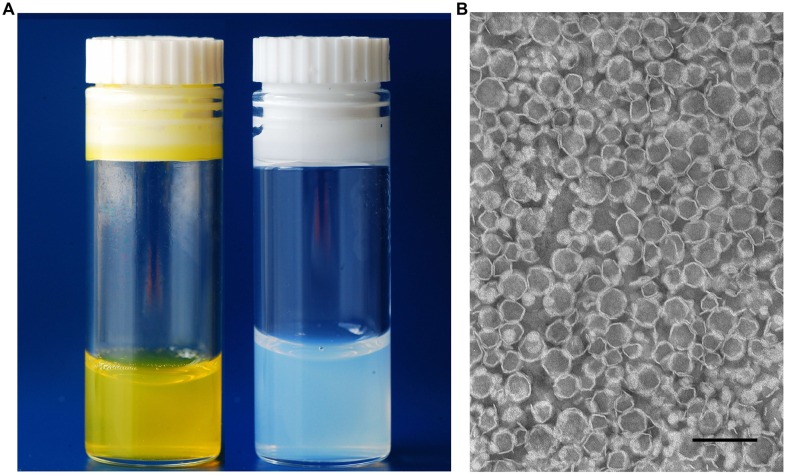
Appearance of liposomal formulations. **(A**) Cur-loaded liposomes **(left)** and free liposomes **(right)**. **(B)** TEM images of Cur-loaded liposomes (H5 formulation). Scale bar represents 200 nm.

The mean diameter size, PDI and zeta potential of the resulting formulations were determined and are summarized in Table 2. The sizes of Cur-loaded liposomes were in the range from 93 to around 112 nm, with a narrow size distribution (PDI), ranging from 0.028 to 0.098 for all formulations. For selected formulations, the liposomes zeta potential was measured and ranged from -2.1 to -3.5. The appearance of Cur-loaded liposomes had a transparent yellow color, without any existence of crystal as shown in Fig 1A (left). The TEM of the most stable H5 formulation revealed that the Cur-loaded liposomes had a homogeneous size and irregular spherical shape (polygonal-like), as shown in Fig 1B.

**Table 2:** Characteristics of the Cur-loaded liposomal formulations.

Formulations	Size (nm)	PDI	Zeta potential (mV)	IE(%)
H5	101 ± 1.2	0.046 ± 0.031	-3.5 ± 0.2	96 ± 2
H10	101 ± 2.1	0.049 ± 0.006	-2.1 ± 1.4	93 ± 3
H12	093 ± 1.1	0.043 ± 0.011	-	89 ± 1
H15	096 ± 1.5	0.037 ± 0.013	-	73 ± 2
HCh10	113 ± 1.2	0.028 ± 0.022	-	35 ± 5
SCh5	099 ± 1.2	0.065 ± 0.015	-2.7 ± 0.7	92 ± 5
SCh10	103 ± 1.7	0.065 ± 0.012	-2.1 ± 0.3	85 ± 2
SCh12	100 ± 1.4	0.063 ± 0.012	-	49 ± 3

### Determination of the incorporation efficiency with different curcumin-to-lipid ratios

**Fig 2:**
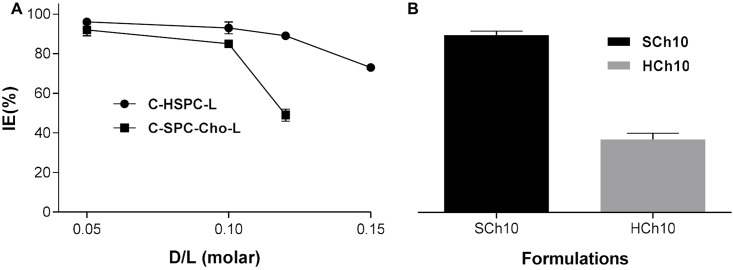
**Incorporation efficiency (IE) of** Cur in different liposome-types (A). Comparison of the incorporation efficiency between C-HSPC-Cho-L (HCh10) and C-SPC-Cho-L (SCh10) formulations when 10% molar of Cur was added (B). The results are shown as the mean ± SD of three replicates.

The incorporation-efficiencies of Cur incorporated into liposomal bilayer were tested using different drug-to-lipid molar ratios for different lipid compositions. High (> 85%) incorporation efficiency could be achieved for a drug-to-lipid molar ratio of up to 0.1, and it decreased with increasing Cur-to-lipid ratios for all formulations. The results are summarized in Fig 2 and are also presented in Table 2. Four formulations were selected for in vitro studies, based on achieving a Cur IE (%) higher than 85% and good stability, namely formulations H5, H10, SCh5 and SCh10.

### Stability of curcumin in liposomes—preliminary studies

**Fig 3:**
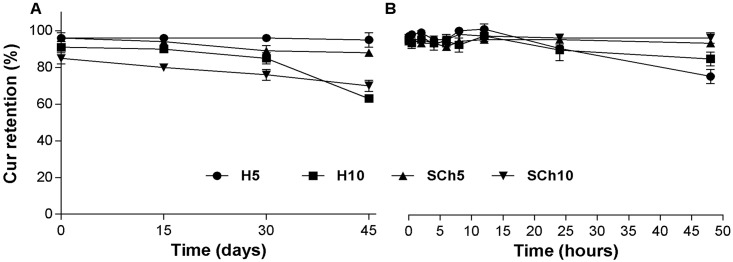
Stability of Cur-loaded liposomes over different storage times and temperature. Comparison of the stability of H5, H10, SCh5 and SCh10 formulations at 4°C (**A**) and at 37°C (**B**). The results are shown as the mean ± SD of three replicates.

No crystal formation was observed in the selected formulations during any of the storage times except at day 45 for the H10 formulation, where yellow Cur crystals were observed. Fig 3 shows that all selected formulations of Cur-loaded liposomes had a desirable stability, although there was a slightly decreased, though not statistically significant Cur retention (%) over the time-period. All formulations were stable for 30 days at 4°C and for 24 hours at 37°C.

### In vitro release of curcumin from liposomes in the presence of human plasma

**Fig 4:**
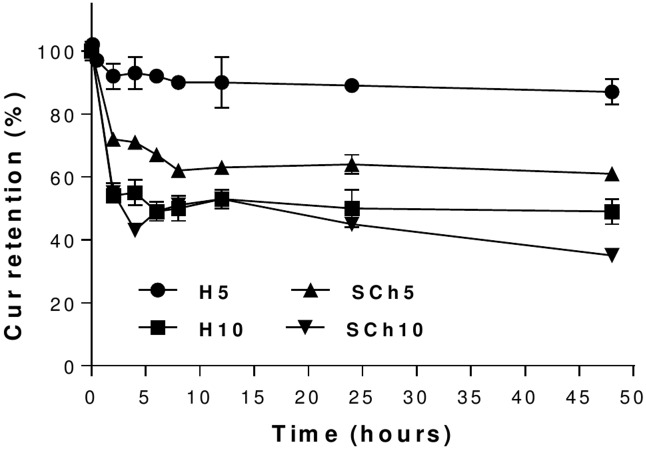
The plasma stability of curcumin-loaded liposomes for the H5, H10, SCh5 and SCh10 formulations. The results are shown as the mean ± SD of three replicates.

Incubation of Cur-loaded liposomes with human plasma resulted in a release of Cur. This was minimal in the case of the H5 formulation, which was found to exhibit higher stability in human plasma among all formulations. There was 13% release of the Cur from the H5 formulation after 48 hours of incubation, while H10, SCh5 and SCh10 formulations showed release of 51, 39 and 65% of Cur, respectively (Fig 4).

### Effect of curcumin-loaded liposomes on the viability of pancreatic and normal cell lines

**Fig 5:**
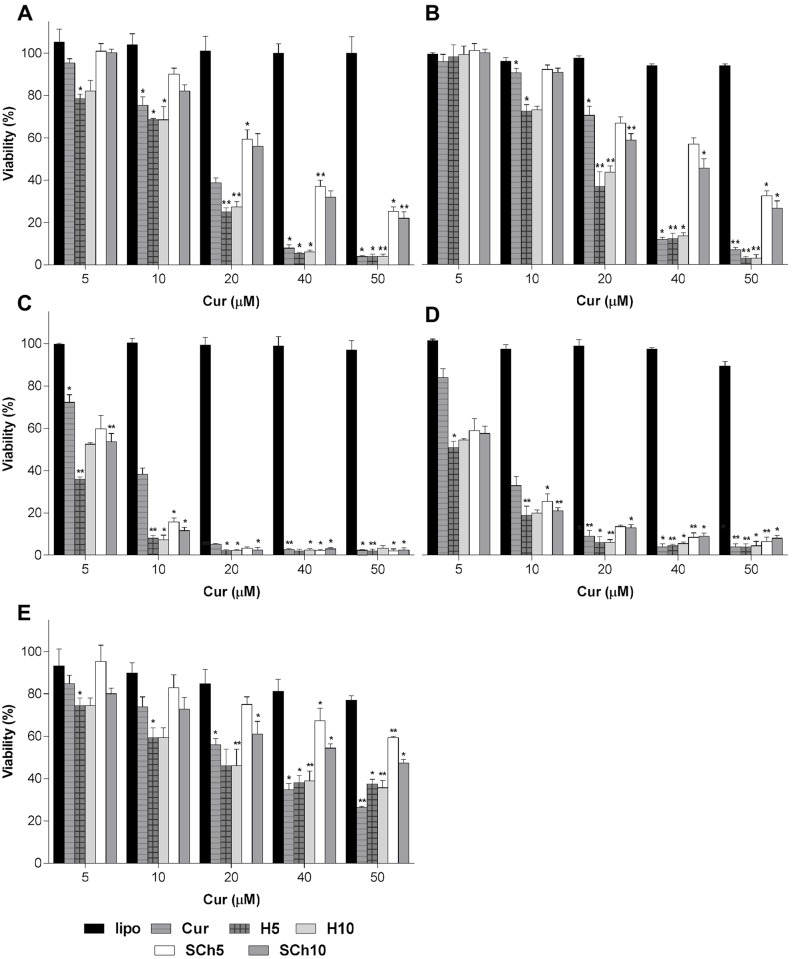
Cytotoxicity of free Cur, free liposomes, H5, H10, SCh5 and SCh10 formulations on AsPC-1, BxPC-3 and NDHF cells using MTT and ATP assays for 72 hours. **(A)** AsPC-1 and MTT, **(B)** AsPC-1 and ATP, **(C)** BxPC-3 and MTT, **(D)** BxPC-3 and ATP; and **(E)** NDHF and MTT. The data are shown as the mean ± SD of three independent replicates. Statistical analysis by unpaired student *t*-test: *p<0.05; **p<0.001.

The cytotoxicity of the selected formulations was tested over a 72-hour period using two different viability assays (MTT and ATP) on AsPC-1, BxPC-3 and normal NHDF cell lines. The results from the concentration-dependent (5–50 μM) treatment of selected Cur formulations, including both free Cur and Cur in the different liposomal formulations were slightly different between the assays (with the ATP assay more sensitive than MTT), as shown in Fig 5 and the IC50 data in Table 3, for instant, the H5 formulation, which contains HSPC lipid and 5% molar curcumin, had the best toxicity profile towards the AsPC1 cell line (IC50 of MTT and ATP, 9.46 and 14.72 μM, respectively), and BxPC-3 cell line (IC50, MTT and ATP, 9.46 and 14.72 μM, respectively). The cytotoxicity differences between the formulations, as determined by the ATP assay, were H5>H10>SCh10>SCh5>Free Cur in both cell lines. While there were only minor difference between the effects of the formulations on the BxPC-3 cell line (IC50, ATP, 6.62, 3.37, 3.67, 3.67, 4.34 and 6.62), there were large and significant differences between the effects of the formulations in the case of AsPC-1 (IC50, ATP, 41.01 and 33.95, for SCh5 and SCh10, respectively; 14.72 and 15.92, for H5 and H10 formulations, respectively). As for the normal cell line, the cytotoxicity of the selected formulations (H10>H5>SCh10>SCh5) shown in the MTT assay was less than that for free Cur (Fig 5E). Further experiments to determine ROS, 3/7 caspase activity and detection of apoptosis were performed using the best formulation in term of the highest IC50 value shown in the ATP assay, namely H5, and compared with free Cur.

**Table 3:** Comparison of IC50 values of cytostatic agents using MTT and ATP Assays on BxPC-3 and AsPC-1 cells.

	BxPC-3		AsPC-1	
Formulations	MTT	ATP	MTT	ATP
Cur	5.84	6.62	14.6	22.82
H5	1.92	3.37	9.46	14.72
H10	2.1	3.67	10.52	15.92
SCh5	3.4	4.76	30.91	41.01
SCh10	3.18	4.34	25.56	33.95

### Effect of curcumin-loaded liposomes on ROS generation

**Fig 6:**
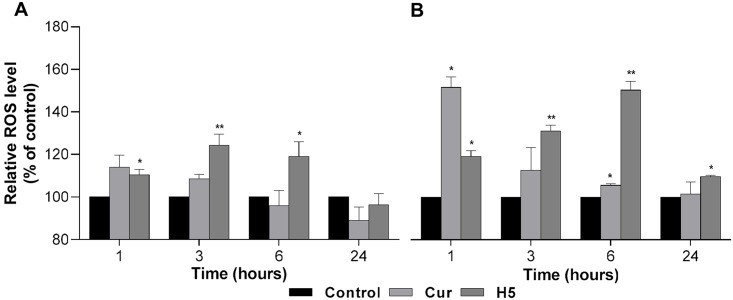
Effect of Cur treatment on ROS generation. Intracellular ROS concentration was determined by treating AsPC-1 **(A)** and BxPC-3 **(B)** cells with free Curcumin and Curcumin in the H5 formulation, with Cur used at the effective IC50 concentration at time intervals of 1, 3, 6 and 24 hours, respectively. The data are shown as the mean ± SD of three independent replicates. Statistical analysis by unpaired student *t*-test: *p<0.05; **p<0.001.

Intracellular ROS can decrease proliferation and induce apoptosis in cancer cells [31]. In order to determine whether the reduction in cell viability that was observed from the treatment of the ASPC-1 and BxPC-3 cell lines with free Cur or the H5 Cur formulation was caused by apoptosis, the generation of ROS was determined. Interestingly, the generation of ROS in both PA cell lines increased significantly in a time-dependent manner, as shown in Fig 6. The ROS level in the case of the AsPC-1 cell line treated with free Cur was 1.14-fold higher after one hour and decreased gradually to 1,09-fold after 3 hours, while for the H5 formulation, the ROS level increased initially and then decreased over time (1.10, 1.24 and 1.19-fold at 1, 3 and 6 hours, respectively) (Fig 6A). In the case of the BxPC-3 cell line, the level of ROS increased 1.52-fold after an hour and then decreased with time to 1.13 and 1.06-fold after 3 and 6 hours, respectively, whereas for the H5 formulation, the ROS level was higher even after 24 hours (1.2, 1.31, 1.5 and 1.1-fold after 1, 3, 6 and 24 hours respectively) (Fig 6B).

### Effect of curcumin-loaded liposomes on caspase 3/7 activation

**Fig 7:**
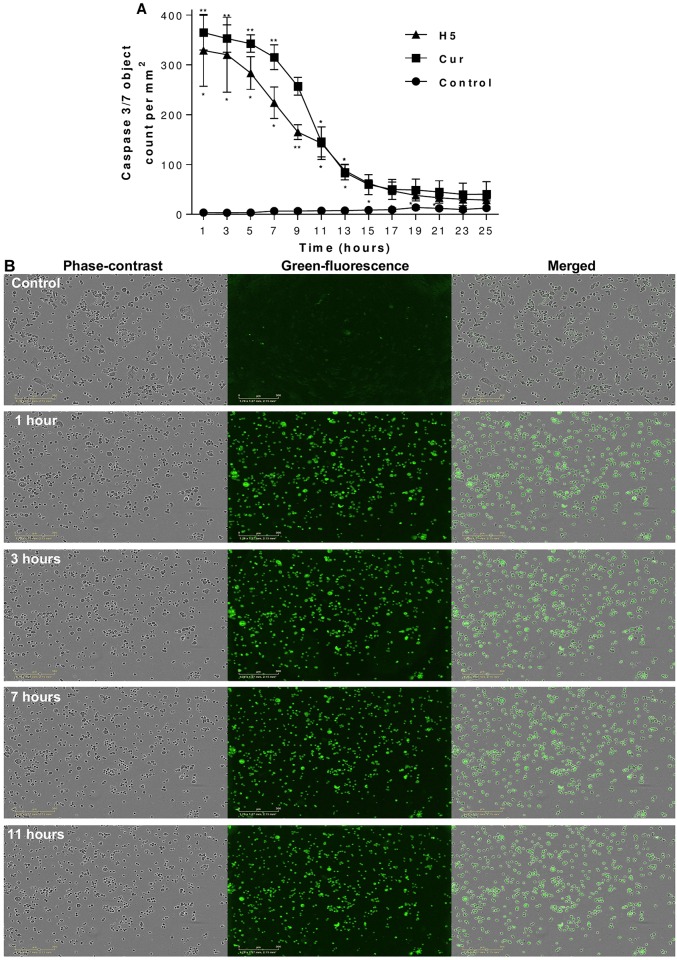
Effect of curcumin treatment on caspase-3/7 activity and morphological changes in AsPC-1 cells. AsPC-1 cells were treated with free curcumin and the H5 formulation containing curcumin, with the effective curcumin concentration equivalent to the IC50 in both cases. Caspase-3/7 activity **(A)**, with morphological changes in real-time using phase-contrast and green fluorescence images showing apoptosis **(B)**. The data are shown as the mean ± SD of three independent replicates. Statistical analysis by unpaired student *t*-test: *p<0.05; **p<0.001. Scale bar represents 300 μm.

**Fig 8:**
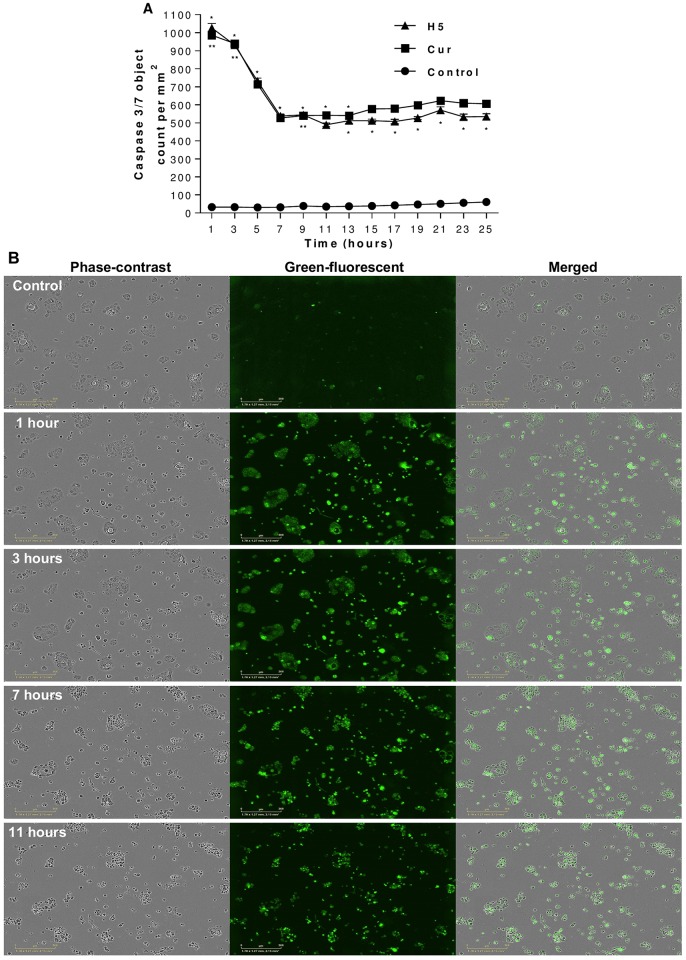
Effect of curcumin treatment on caspase-3/7 activity and morphological changes in BxPC-3 cells. BxPC-3 cells were treated with free curcumin and the H5 formulation containing curcumin, with the effective curcumin concentration equivalent to the IC50 in both cases. Caspase-3/7 activity **(A)**, with morphological changes in real-time using phase-contrast and green fluorescence images showing apoptosis **(B)**. The data are shown as the mean ± SD of three independent replicates. Statistical analysis by unpaired student *t*-test: *p<0.05; **p<0.001. Scale bar: represents 300 μm.

The activation of caspase 3/7 results in the irreversible commitment of a cell to apoptosis. Cells were treated with IC50 of free Cur and H5 formulation. Caspase 3/7 activity was shown to be significantly increased in both PA cell lines in a real time by treatment as shown in Figs 7A and 8A. In addition, the green fluorescence images of caspase 3/7 assay confirm the presence of cells which have undergone apoptosis (Figs 7B and 8B). The green fluorescence related to caspase 3/7 activation was at its highest point after one hour of treatment and decreased gradually after some time for both PA cell lines.

### Morphological changes and apoptosis detections

Morphological changes were monitored under phase-contrast microscopy. Figs 7B and 8B show significant morphological changes in the majority of AsPC-1 and BxPC-3 cells, when exposed to Cur-loaded liposomes in real-time images, compared with control. Treated cells exhibited rounding, cytoplasmic blebbing, shrinking and irregularity in shape which is typically a sign of apoptosis. Also, a reduced viable cell-count was observed over the time-frame of the experiment.

**Fig 9:**
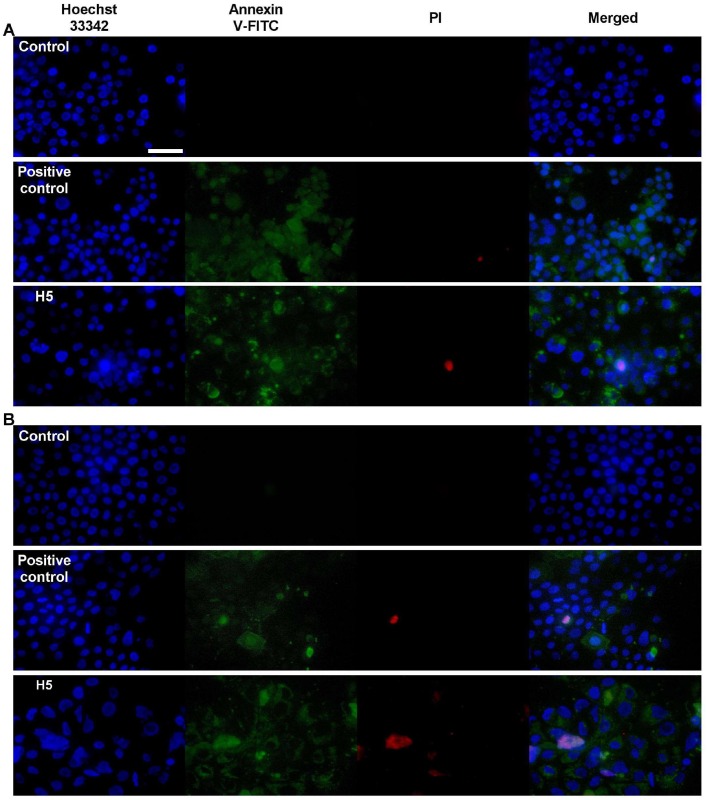
Detection of apoptotic cells using annexin V-FITC and PI double-staining. AsPC-1 cells **(A)** and BxPC-3 cells **(B)** were treated with curcumin in the H5 formulation at a concentration equivalent to the IC50 of curcumin for 72 hours. Early (annexin V-FITC staining) and late (PI staining) apoptotic stages are shown. Scale bar represents 50 μm.

Finally, membrane phosphatidylserine externalization was observed, which indicates the presence of apoptotic cells. As shown in Fig 9A and 9B, early and late apoptotic cells were observed for both PA cell lines when exposed to the treatments with the Cur and Cur-loaded liposomal formulations.

## Discussion

This study is the first to summarize the incorporation efficiency, stability at different temperatures, *ex vivo* plasma stability, anticancer activity towards PA of several Cur liposomal formulation; and finally the ability of Cur-loaded liposome to induce apoptosis by both ROS generation and activation of caspase 3/7 in PA cell lines. These liposomes were designed for PA metronomic therapy in order to select the formulation with highest stability, high antitumor activity and having the potential to deliver Cur to the cancer tissue by EPR effect. Among them, a cholesterol free, PEGylated formulation, composed from hydrogenated soya PC (HSPC), is reported for the first time (termed H5, containing HSPC, DSPE-PEG_2000_ and Cur (9.0:0.5:0.5 molar ratio, respectively) as an interesting alternative to the previously reported formulations, most of which were charged, conventional and non-long circulating with could not contribute to the increased accumulation in the cancer tissue via the EPR effect. The H5 formulation was found to have a high stability *in vitro*, low drug release in plasma *ex vivo* and the highest cytotoxic activity for pancreatic cancer cell lines, as demonstrated by its ability to induce apoptosis by both ROS generation and activation of caspase 3/7 in the two PA cell lines tested. In contrast, liposomes composed from soya phosphatidylcholine (SPC) showed an antagonistic effect with Cur. This undermines the rationale of the use of this lipid for Cur encapsulation for PA treatment.

The physicochemical characterization of liposomes, such as size, shape and charge are vital parameters to deliver improved bio-distribution and prolonged pharmacokinetics of encapsulated cytotoxic drugs [32,33] Table 2 summarizes the physicochemical parameters of all of the tested liposomal formulations. The preparations had a small homogeneous size, low PDI index, indicating good homogenization of liposomes during extrusion, and no aggregation or fusion processes occurring after liposome preparation. In some cases, the zeta-potential of liposomes was determined and the tested liposomes were characterized by a low residual negative charge, possibly derived from charged DSPE-PEG_2000_ molecules which as ours results indicate has no influence on the such liposomes circulation time [34]. Additionally, most liposome types had high incorporation efficiency for Cur.

In our work, we used various lipid compositions (Table 1) (HSPC and SPC) to prepare Cur-loaded liposomes. In addition, cholesterol was added to modulate the fluidity/rigidity of liposomes membranes. DSPE-PEG_2000_ was added to modify the surface of liposomes in order to have a formulation exhibiting a prolonged circulation time, so as to improve liposome accumulation in cells and potentiate the EPR effect. The C-HSPC-L formulation showed the ability to incorporate with 89% of Cur when a drug-to-lipid molar ratio of 0.12/10 (H12 formulation) was used (Fig 2A). Addition of cholesterol to the HSPC/Cur liposomal preparations (H10 vs HCh10) caused a dramatic decrease in the incorporation efficiency of Cur (from 93 to 35%). In contrast, when cholesterol was added to an SPC liposomal formulation, an incorporation efficiency of 85% was obtained. Other studies have shown that the presence of cholesterol may compete with highly lipophilic drugs, such as paclitaxel [35]. Since a HSPC bilayer has a high transition temperature and exists in the gel-phase at room and body temperature, it has a stronger order parameter, in comparison to a bilayer composed from a much more fluid SPC. The addition of cholesterol decreases the amount of empty space within the bilayer more dramatically in HSPC than in SPC liposomes [36,37]. The decrease in Cur incorporation efficiency (IE %) observed in the cholesterol-containing HSPC formulation may be associated with such a phenomenon (Fig 2B). The TEM image (Fig 1B) of the best liposomal formulation tested, namely H5, shows spherical liposomes with slightly polygonal shape. This phenomenon is observed in the case of liposomes composed from phospholipids with fully-saturated fatty-acids in the molecule (DPPC, DSPC, HSPC) [38,39]. The addition of cholesterol smooths the surface of liposomes but, as discussed above, it decreases the incorporation efficiencies of most hydrophobic substances. Such cholesterol-depleted liposomes have been demonstrated by Dos Santos et al. to display a very short circulation-time *in vivo* due to rapid opsonisation. However, the addition of even a low level of PEGylated lipids (1–2% only) contributes to making them more stable, and long circulating, thereby fully enabling them to carry drugs for many hours in the blood stream [40,41]. In the same study, Dos Santos et al. concluded that, for hydrophobic drugs, the bilayer rigidity has a pivotal importance in terms of avoiding untimely drug-release. These authors demonstrated that DSPC liposomes depleted of cholesterol were more efficient in retaining the hydrophobic drug, idarubicin, than formulations containing cholesterol. It appears that, when cholesterol is added to rigid HSPC liposomes, it fluidizes the lipid bilayer, driving the release of incorporated drug [40,41].

All Cur-loaded liposomes showed a desirable stability in size and PDI (data not shown, the size and PDI were the same over time) and in IE % in both storage time (at 4°C and 37°C) (Fig 3A and 3B), suggesting that liposomes could serve as a suitable carrier for Cur. Phospholipids with a higher phase-transition temperature, such as HSPC, with a phase transition temperature TM of nearly 50°C, have higher membrane stability and lower drug-release rates, compared with lipids with lower phase-transition temperatures, such as SPC, with a TM below 0°C [11,42] In the present study, the in vitro drug release rates from liposomes with different compositions showed that the HSPC-containing H5 formulation had a high in vitro stability in plasma (13% release after 48 hours.), while H10, SCh5 and SCh10 formulations showed release of 51, 39 and 65% of Cur, respectively (Fig 4). Similar results have been shown by others for brucin and paclitaxel encapsulated in HSPC liposomes, which had a lower release rate than from SPC liposomes [42,43]. As a result, this data supports the concept that a good pharmacokinetic effect in vivo can be achieved using liposomal formulations with high membrane stability. Also, our finding that HSPC liposomes with a higher ratio of Cur (H10) showed a 4-fold higher release rate of Cur than the H5 formulation, which had a lower Cur-to-lipid-ratio, suggests that increasing the ratio of hydrophobic drugs into liposome formulations increases the release-rate of the drug as well as decreases the stability of the liposomal formulation. The initial high drug-release from practically all the formulations could be triggered by proteins absorbing to the liposomal bilayers resulting of the Cur molecules release. This effect was lowest for the H5 formulation which, in principle had the more stable structure. Despite the lower content of Cur within the H5 formulation, and, therefore, lower initial drug release, it may also be easier to deliver it into cancer tissue by the EPR effect since Cur release in plasma for this particular formulation was remarkably slower, compared to other formulations, thereby favoring an increased retention-time *in vivo*. This has an important consequence in achieving a therapeutic effect in such fibrotic cancers as PA. Therefore, the low drug-to-lipid ratio in the case of certain hydrophobic drugs can be regarded as an advantage. As discussed, liposome formulation H5 had the highest stability in 50% human plasma at 37°C, compared with the formulation composed of SPC with the higher drug-to-lipid- ratio. Upon injection, these SPC formulations will be characterized by faster Cur leakage and, probably, a lower Cur accumulation in the cancer tissue. Secondly, the injection of the same dosage of Cur from the liposomes with the decreased drug-to-lipid ratio means a higher lipid dose (two times higher). This, at the first glance, seems to be a disadvantage, but in turn, an increased dosage of liposomes is correlated with a longer circulation time. Saturated lipid doses are correlated with an increased circulation time of the liposomes, even for stealth liposomes [44,45]. Therefore, this can be correlated with a higher probability to accumulate an increased amount of the drug locally within the tumor, by means of the EPR effect.

The cell lines used for the current study were selected according to a review by Emily et al. who summarized the available information as an aid for researchers to select appropriate cell lines for particular research needs [46]. Cur has anticancer activity against different cancers, including pancreatic cancer, which has a particularly high mortality rate. Taking the advantages (namely, anticancer activity) and seeking to overcome the disadvantages (namely, toxicity or solubility) is one approach for scientists to improve drug loading, bioavailability and activity, along with reducing cytotoxicity to normal cells, in order to cure proliferative diseases such as tumors. Taken together, the aforementioned advantages, liposomal formulations of liposomes and Cur showed a significantly increased cytotoxicity, which is probably due to better bioavailability. Cur-loaded liposomes showed a better cytotoxic activity compared with free Cur towards two PA cell lines, as shown in Fig 5A, 5B, 5C and 5D, and in Table 3. These results are comparable with the study of Lan Li et al. [12] which showed a cytotoxic activity of Cur-loaded liposomes towards the PA cell lines, but in our case, different Cur-loaded liposome formulations were used. Engin Ulukayashould et al. mentioned the choosing of drug may be a critical point in the reliability of the cytotoxicity assays [47]. Therefore, two different cytotoxicity assays were used in the present study, with both the MTT assay and the ATP assay showing a reasonably good correlation in vitro cytotoxicity for our formulations.

Moreover, lipids such as sphingosylphosphorylcholine have an anticancer activity of their own towards PA [48]. In our case, free SPC liposome showed cytotoxic activity against the AsPC-1 cell line (Data not shown) and free Cur also had anticancer activity, as shown in the data. Taking all of these considerations together, since free SPC liposomes and free Cur both had cytotoxic activity on ASPC-1 cells, it might be anticipated that loading Cur into SPC liposomes might show a better anticancer activity, but Cur-loaded SPC liposomes actually showed the opposite effect, namely, a 2.7-fold less toxicity compared with C-HSPC-L formulation or free Cur. The two formulations of SCh5 and SCh10 which contained SPC lipids with a different drug-to-lipid ratio, showed a comparable level of cytotoxic activity (SCh5, IC50: 41.01 μM < SCh10, IC50: 33.95 μM, Data in Table 3). It was determined that the SCh10 formulation had less SPC but more Cur (1.0 and 5.5 drug-to-lipid ratio molar), and SCh5 had more SPC but less Cur (0.5 and 6.0 drug-to-lipid ratio molar), which suggests that increasing the ratio of SPC lipids decreases the cytotoxic activity of the formulation, suggesting a possible antagonism between SPC and Cur.

Additionally, lower cytotoxicity of Cur-loaded liposome was observed for normal (NHDF) cells, which requires explanation. It is generally expected that Cur will be more toxic to tumor than normal tissues. Indeed, it was previously reported that Cur induces apoptosis via ROS generation in malignant cells, but not in normal cells [46]. Cur binds to more than 40 different proteins and exerts a profound influence on many cancer cell pathways, modulates growth of tumor cells through regulation of multiple cell-signaling pathways [49,50]. Curcumin inhibits the activation of the NF-κB pathway and the expression of various oncogenes regulated by NF-κB, leading to apoptosis of the cancer cells [51]. Although, Cur-loaded liposomes showed a lower toxicity toward normal than cancerous cells in this study, the toxicity is still present. Another explanation of the lower activity of Cur-loaded liposomes toward normal cells could be related with the levels of iron ions in the cells, which is often higher in cancer than in normal cells, due to overexpression of the transferrin receptors. Higher levels of iron generate higher level of ROS in cancer cells. The higher levels of Cur acts in a pro-oxidative manner and increases the already high level of ROS in the cancer cells, leading to cell death by apoptosis [52,53]. Therefore, the monitoring of apoptosis in response to Cur treatment was particularly investigated in our study on PA.

Apoptosis, or programmed cell death, is a normal physiological process which has been characterized by various biochemical and morphological changes including cell shrinkage, chromatin condensation, membrane budding, protein fragmentation and DNA fragmentation, appearance of apoptotic bodies and activation of specific proteases, so-called caspases [50,54]. Generally, there are two major pathways of apoptosis; an intrinsic one, which involves in activation of aspartate-specific cysteine protease (caspases) and an extrinsic one which involves the engagement of death receptors (tumor necrosis factor family). However, the failure of cells to fully undergo apoptosis has been involved in tumor development and is part of the mechanism associated with tumor cell resistance to cancer therapy. Cur has been demonstrated to be involved in initiating both caspase-dependent and caspase-independent apoptosis pathways in cancer [50,55] Previous studies have shown that Cur can induce apoptosis by activation of caspase 3 or ROS generation, which leads to multiple apoptotic signals, including caspase-dependent and caspase-independent pathways in colon cancer [55]. Our results demonstrate that Cur-loaded liposomes have a significant ability to generate intracellular ROS in a time-dependent manner, even after 6 hours and 24 hours for the AsPC-1 and BxPC-3 PA cell lines, respectively. This is an important observation, since this study shows that both these mediators of apoptosis are affected by Cur treatment and that these effects are at least twice as long compared to the same effects obtained with free Cur, which were 3 hours and 6 hours for AsPC-1 and BxPC-3 cell lines, respectively (Fig 6A and 6B). From these results, we suggest that Cur-loaded liposomes can protect Cur from degradation. Wang et. al showed 20% and 50% of free Cur decomposed within 1 and 8 hours respectively after incubation at 37°C in cell culture medium containing 10% fetal calf serum [56]. Also, it is possible that Cur-loaded liposomes can effectively deliver Cur into pancreatic cancer cells and induce apoptosis by prolonging the generation of intercellular ROS. This effect can be additionally explained by the fact that liposomal Cur is mostly in the form of free molecules, whereas added as a solution in DMSO, it can be prone to partial crystallization (especially at higher concentrations) leading to decreased drug bioavailability.

Also, Cur-loaded liposomes showed the ability to induce apoptosis via the activation of caspase 3/7 within one hour of exposure to the treatment in both cell lines (Figs 7A, 7B and 8A, 8B). The inhibition of caspase 3/7 is related with failure of ATP generation [57] which our results strongly support. Apoptosis induction in AsPC-1 and BxPC-3 PA cell lines was associated with morphological changes (cytoplasmic blebbing, shrinking and irregularity in shape and the externalization of cell membrane phosphatidylserine) which were preceded by an increase in intracellular ROS generation and activation of caspase 3/7. Our studies have demonstrated that Cur induces apoptosis via both caspase dependent and independent pathways in pancreatic cancer cell lines and our findings are supported by many previous studies in PA and different cell lines [12,50,55,57–59].

Numerous approaches have been undertaken to encapsulate Cur within liposomes, but most of them utilize fluid or charged liposomes with short pharmacokinetics. Therefore, our goal was to establish a new stable, rigid formulation of Cur with excellent in plasma retention ability and a formulation having excellent anticancer properties. Those liposomal formulations might be able to reach PA if injected repetitively several times using a metronomic approach or in combination with other chemotherapeutics, preferably in the form of nanocarriers. Therefore, our studies are the first step in demonstrating the utility of a new approach to pancreatic cancer therapy involving long-circulating Cur-containing liposomes which might be able to not only increase the bioavailability of the therapeutic agent, but also to have a sufficiently long biological retention time to enable the accumulation of the liposomes in cancer tissue by the EPR effect.

Taking into consideration that Cur exerts less activity on healthy cells, prolonged in vivo injection of such liposomes with a concurrent long-term therapeutic effect could be possible, in principle. In further studies, this formulation H5, with PEGylated lipids will be tested in an animal model, to test the rationale of our approach.

## Conclusion

In conclusion, our results showed that H5 Cur-loaded liposomes had a desirable stability and potent anticancer activity in pancreatic cancer cell lines and demonstrated a lower cytotoxicity on a normal cell line. Cur-loaded liposomes induced apoptosis through generation of intracellular ROS and activation of caspases 3/7. Future studies are warranted to further explore the potential of this Cur-loaded liposome delivery system as an independent pancreatic cancer therapy, or in combination with other drugs. Systems based on the use of phospholipids, such as SPC, can be regarded as an interesting platform to deliver the incorporated substances into the bloodstream, but in this case these liposomal formulations show low stability in plasma *ex vivo* and an antagonistic effect between Cur and SPC, which diminishes the anticancer potential of Cur, leading to at least partial questioning of this lipid as a carrier for Cur for PA cancer treatment.
