# Correction: Long-Circulating Curcumin-Loaded Liposome Formulations with High Incorporation Efficiency, Stability and Anticancer Activity towards Pancreatic Adenocarcinoma Cell Lines *In Vitro*

**DOI:** 10.1371/journal.pone.0173728

**Published:** 2017-03-08

**Authors:** Mohamed Mahmud, Adriana Piwoni, Nina Filipczak, Martyna Janicka, Jerzy Gubernator

The third author’s name is spelled incorrectly. The correct spelling is: Nina Filipczak. The correct citation is: Mahmud M, Piwoni A, Filipczak N, Janicka M, Gubernator J (2016) Long-Circulating Curcumin-Loaded Liposome Formulations with High Incorporation Efficiency, Stability and Anticancer Activity towards Pancreatic Adenocarcinoma Cell Lines *In Vitro*. PLoS ONE 11(12): e0167787. doi:10.1371/journal.pone.0167787
